# The apheresis platelet donation was increased after a nationwide ban on family/replacement donation in China

**DOI:** 10.1186/s12889-021-10819-4

**Published:** 2021-04-29

**Authors:** Jinyan Chen, Guoli Zhou, Xuemei Fu, Shijie Li, Ying Li, Jianxun Kang, Huiyou Chen, Liqiao Zhou, Yongshui Fu

**Affiliations:** 1 Guangzhou Blood Center, 31 Lu Yuan Road, Guangzhou, Guangdong China; 2 Clinical and Translational Sciences Institute (CTSI), Michigan State University, 909 Wilson Road Suite B500, East Lansing, MI 48824 USA; 3 Chengdu Blood Center, Chengdu, Sichuan China; 4 School of Laboratory Medicine and Biotechnology, Southern Medical University, Guangzhou, Guangdong China

**Keywords:** Plateletpheresis donation, Ban on family/replacement donation, Pseudo-panel data approach, Piecewise linear mixed model

## Abstract

**Background:**

A nationwide ban on family/replacement donation (FRD) went into effect on April 1, 2018 in China. To date, no reports relevant to the trend of plateletpheresis donations before and after a nationwide ban on FRD were found.

**Methods:**

We used two independent full samples, consisting of 135,851 and 82,129 plateletpheresis donors from Guangzhou and Chengdu between October 2012 and September 2019, respectively. A pseudo-panel data approach was applied by grouping three time-invariant covariates – gender, blood donation history, and birth year across 14 cross-sections (a 6-month interval each) to form a total of 24 cohort groups (14 × 24 = 336 cohorts, i.e., cells) with each having common covariates. The outcome was average apheresis platelet units per donor in each cell. We performed a two-piecewise linear mixed model with the cross-section (i.e., time) just right before the ban as a time breakpoint (i.e., 11th cross-section) to examine the trend of outcome with the adjustment of three time-invariant covariates. We removed the FRDs in each of the first 11 cross-sections to detect its possible influence on the trend.

**Results:**

The final model for the samples from Guangzhou presented a two-piecewise linear trend of the outcome over time with a horizontal line to the left of the breakpoint (β_timeBefore11_ = 0.0111, *p* = 0.0976) and a significantly positive linear trend to the right (β_timeAfter11_ = 0.0404, *p* < 0.0001). The male donors and the donors with plateletpheresis donation history had an increased baseline outcome and a significant outcome change over time after the ban. Such a two-piecewise linear trend pattern can be replicated using the samples from Chengdu with some minor variations. Removing the FRD before the ban can change the pattern.

**Conclusion:**

The significant increase of the average apheresis platelet units per donor over time after the FRD ban may be related to the implement of the FRD ban and the improved donation behavior of male donors and/or donors with platelet donation history after the ban. Our findings may potentially motivate the policymakers in other countries where the FRD for plateletpheresis donation is still legitimate to phase out their FRD strategy and ultimately achieve 100% voluntary plateletpheresis donation.

**Supplementary Information:**

The online version contains supplementary material available at 10.1186/s12889-021-10819-4.

## Background

The adequacy and safety of blood supply is a major public health challenge in the world [1, 2]. There is an ongoing debate over the family/replacement donation (FRD) policy influencing both shortage and safety of blood supply [3–5]. FRD, also called mutual donation, occurs when family members are required to donate blood to replace each unit used by their friend or relative [6]. Currently, the FRD is legitimate and considered to be indispensable to the transfusion services in many countries with the limited resources [7–9]. Although this type of blood donation may provide short-term solutions for dealing with the shortage of blood supply [10], it increases public distrust in voluntary blood donation and affects the quality and safety of donated blood [11] . Phasing out the FRD is one of the targets in a global framework for action to achieve 100% voluntary non-remunerated blood donation (VNRBD) developed by the World Health Organization and the International Federation of Red Cross and Red Crescent Societies [9].

A nationwide ban on the FRD went into effect on April 1, 2018 in China. With the “more donors, more blood “ belief, it was believed that banning the FRD would cause serious consequences related to the shortage of blood supply [8, 12]. However, a phenomenon of “fewer donors, more blood” for the plateletpheresis donation has been emerging in Guangzhou Blood Center and Chengdu Blood Center since the implement of the FRD ban in China, even though there is no substantial difference in the publicity and recruitment of blood donation between before and after the FRD ban in the two blood centers. To date, no reports relevant to the trend of plateletpheresis donations before and after a nationwide ban on family/replacement donation were found. Therefore, it is necessary to quantify the evidence-based trend of the plateletpheresis donation and the potential contributing factors to this trend before and after the FRD ban in China.

In present study, we examined a model-based trend of the apheresis platelet units donated before and after the FRD ban using two independent full samples in China with a pseudo-panel data approach followed by a piecewise linear mixed model.

## Methods

### Study population and study design

Based on the availability of the data, we used two independent full samples between October 1, 2012 – September 30, 2019 from Guangzhou Blood Center, the second largest blood center in China and Chengdu Blood Center, the largest blood centers in Western China, which were named as “GZ set” and “CD set” at the individual level, respectively. The GZ and CD sets consisted of 135,851 and 82,129 plateletpheresis donors, respectively. We enrolled the donors with an age of 18 ~ 60 years old, weight ≥ 50 kg for male and ≥ 45 kg for female, systolic blood pressure 90 ~ 140 mmHg and diastolic blood pressure 60 ~ 90 mmHg, pulse 60 ~ 100 beats/min, normal body temperature, platelet counts before donation 150 ~ 450 × 10^9^/L, and no any other health conditions according to the China national standard for the eligible donor selection criteria (GB 18467–2011). All data used in the present study were de-identified.

We chose 5.5 years and 1.5 years before and after April 1, 2018, respectively, to set our investigation time window with a six-month interval as a cross-section, generating a total of 14 repeated cross-sections with 11 and 3 cross-sections before and after the ban, respectively. Based on the GB 18467–2011, a donor may donate up to a total of 24 plateletpheresis collections during a 12-month rolling period. Thus, some plateletpheresis donors may appear more than once across the 14 cross-sections, but not all donors appear in every cross-section. Therefore, there were some different degrees of the correlations of the data across 14 cross-sections, and our datasets presented a pseudo-panel structure [13].

### Measurements of outcome variable and covariates

The records for the total amount of the platelets donated by each donor within each cross-section, grouping covariates – gender, birth year, and blood donation history (see detailed information below), as well as the variable –family/replacement plateletpheresis donation (yes vs. no) were extracted from the archived blood donation documents in both blood centers. When a blood donor came to donate blood and assigned his donated blood to a specific patient on the waiting list, his/her donation was marked as FRD. The outcome variable at the cohort (i.e., cell) level within the pseudo-panel datasets (see detailed information below) was defined as average plateletpheresis units per donor in each cell.

### Construction of pseudo-panel datasets

The pseudo-panel data approach is actually a solution to transform the individual-level cross-sectional data into the group-level data (i.e., pseudo-panel data) such that the typical longitudinal models can be applied to efficiently and consistently estimate the change of the interested outcome variable over time [13]. This approach has been increasingly applied to public health [14, 15].

According to the methods described in the literature [16, 17], we constructed two pseudo-panel datasets from our GZ and CD data by grouping three time-invariant variables - gender, birth year, and blood donation history. Other potential covariates such as ethnicity, occupation, and education with large proportions of missing data were excluded from the analyses. Briefly, individual platelet donors were first classified based on gender that had two categories – male and female. To balance the size of each cohort (≥100, named as large cohort) and the number of large cohorts [16] within our pseudo-panel datasets, we defined birth year as three categories – “1952–1974”, “1975–1984”, and “1985–2001”. The variable blood donation history was coded as 4 levels – “None” (i.e., the blood donor had never donated blood before the current cross-section, also called no history of donations), “Whole Blood Donation Only” (i.e., the blood donor had donated whole blood only before the current cross-section, abbreviated to “WB”), “Apheresis Platelet Donation Only” (i.e., the blood donor had donated apheresis platelet only before the current cross-section, abbreviated to “PLT”), and “Both WB and PLT Donations” (i.e., the blood donor had donated both whole blood and apheresis platelet before the current cross-section, abbreviated to “Both”). The individuals were then further divided by these two variables, generating 2 × 3 × 4 = 24 cohort groups across the 14 cross-sections, i.e., 24 × 14 = 336 cells for each of two pseudo-panel datasets. The generated each cohort group had common gender, birth year, and blood donation history. To reduce the measurement error [17], we removed the cells with less than 30 individual donors in each dataset to generate two final pseudo-panel datasets, named as “overall Guangzhou pseudo-panel set” (abbreviated to “overall GZ set”) (*n* = 330 cells) and “overall Chengdu pseudo-panel set” (abbreviated to “overall CD set”) (*n* = 316 cells), respectively, for further analyses. More detailed information about the summaries of the constructed pseudo-panel datasets is presented in Additional file 7.

Our study was actually an observational study using pseudo-panel approach to analysis the data [13–15]. Thus, we used the Strengthening the Reporting of Observational Studies in Epidemiology (STROBE) (Additional file 1) statement to ensure standardization and enhance the quality of the reporting [18].

### Statistics

For the individual-level data, we used two-tailed independent t-tests (α = 0.05) to compare total number of donors, total number of donations, and total apheresis platelet units (U), average plateletpheresis donations per donor, and average apheresis platelet units (U) per donor between before and after the ban (also called “after-before mean difference”). To compare two after-before mean differences between groups within each covariate, we applied two-tailed Z-test (Z = (mean difference_1_-mean difference_2_)/sqrt (se_1_^2^ + se_2_^2^), α = 0.05).

For overall GZ pseudo-panel set, we plotted outcome values (i.e., average apheresis platelet units per donor per cell) from each cohort group that had common gender, birth year, and blood donation history versus time (i.e., 14 cross-sections) as well as overall average outcome values from all 24 cohort groups versus time to visualize whether the trend of the outcome over time is linear or non-linear. Given that generalized linear mixed model and mixed-generalized ordered logit model have been successfully applied to analyze the trend of the outcome variable (proportion or probability) over time within a pseudo-panel dataset [14, 19], for our pseudo-panel dataset with a continuous outcome variable, we applied a linear mixed model for modelling the linear trend, or modelling the non-linear trend using a piecewise linear mixed model with the defined time (i.e., cross-section) breakpoint(s) based on the above-mentioned visualization [20]. We conducted a model selection starting from an unadjusted model (i.e., model 1 – pure time trend model without any covariates) to an adjusted model (i.e., model 2 = model 1 + significant covariates), and then to a final model (i.e., model 3 = model 2 + significant interaction terms). To compare two raw regression coefficients within the same final model, we used one-tailed Z-tests [Z = abs(β_2_-β_1_)/sqrt (SE_1_^2^ + SE_2_^2^), α = 0.05].

The assumptions of normality and homoscedasticity for piecewise linear mixed effects models were examined by visualizing marginal (for fixed effects only) and conditional (for both fixed and random effects) Pearson residual plots.

For five-fold cross-validation of the final model, the data were randomly split into 5 roughly equal-sized subsets using SAS PROC SURVEYSELECT procedure, the final model was fitted to the 4 subsets of the data using SAS PROC MIXED procedure with the STORE statement. The prediction error – Root Mean Square Error (RMSE) of the fitted model to predict the fifth subset using SAS PROC PLM procedure was calculated. This procedure was repeated 5 times such that each subset was used for testing exactly once and an average RMSE of 5-fold cross-validation was calculated by using the formula: SQRT((RMSE_1_^2^ + RMSE_2_^2^ + … + RMSE_5_^2^)/5) and further compared with that from the model-fitting using the full sample to determine if there was an over-fitting issue.

To test for replication of the trend of the overall outcome mean over time obtained from the overall GZ set, we applied the same methods as described above to the independent pseudo-panel dataset from Chengdu Blood Center, i.e., overall CD set.

To detect the potential effect of family/replacement plateletpheresis donations on the model-based trend of the average apheresis platelet units per donor over time identified from the overall GZ and CD sets, we removed the family/replacement plateletpheresis donations in each of the first 11 cross-sections (all donations after the ban were voluntary) from the overall individual-level data. Then we re-grouped the remaining individuals (81,801 and 48,768 voluntary plateletpheresis donors remaining from the GZ and CD sets, respectively) to generate two nested pseudo-panel subsets, named as “voluntary GZ subset” (*n* = 300 cells) and “voluntary CD subset” (*n* = 284 cells), respectively. Finally, we used the same methods as described above to fit the piecewise linear mixed models for both subsets.

All data management and statistical analyses described above were conducted with R (R Development Core Team) and SAS v9.4 (SAS Institute, Cary, North Carolina).

## Results

### Demographics of plateletpheresis donors across 14 successive cross-sections with a 6-month interval each in both overall GZ and CD sets

Table 1 and Additional file 2 showed a similar demographics of plateletpheresis donors across 14 successive cross-sections between overall GZ and CD sets. Our overall GZ set consisted of 69.3–80.3% of plateletpheresis donors who were male across all 14 cross-sections, 5.0–6.5% and 8.8–9.7% ≥46 years old across the first 11 cross-sections (i.e., before the ban) and the 12th – 14th cross-sections (i.e., after the ban), respectively, as well as 45.1–51.8% of donors who had a blood donation history (14.4–14.5%: WB only, 18.6–21.9%: PLT only, and 12.0–15.5%: Both) across the cross-sections before the ban and 74.8–76.8% with a blood donation history (17.5–20.9%: WB only, 27.9–28.5%: PLT only, and 25.4–31.5%: Both) across the cross-sections after the ban (Table 1). The overall GZ set also contained about 15.4–41.9% of FRDs across the first 11 cross-sections (for the 12th – 14th cross-sections, no donations were FRD) (Table 1). In the overall CD set, about 63.3–75.5% were male across all 14 cross-sections; 10.0–13.9% and 16.3–17.9% were ≥ 46 years old across the first 11 cross-sections and the 12th – 14thcross-sections, respectively; 30.1–40.6% and 63.9–72.4% had a blood donation history across the cross-sections before and after the ban, respectively; and 3.9–52.6% were family/replacement donors across the first 11 cross-sections (Additional file 2).

**Table 1:** Demographics of individual plateletpheresis donors across 14 successive cross-sections in Guangzhou Blood Center (*N* = 135,851)

	2012/10−2013/3	2013/4–2013/9	2013/10–2014/3	2014/4–2014/9	2014/10–2015/3	2015/4–2015/9	2015/10–2016/3	2016/4–2016/9	2016/10–2017/3	2017/4–2017/9	2017/10–2018/3	2018/4–2018/9	2018/10–2019/3	2019/4–2019/9
Gender														
Male, n (%)	5409 (71.0)	6176 (71.5)	5944 (69.3)	6938 (71.3)	7371 (72.1)	8117 (73.0)	8395 (73.3)	8968 (77.9)	8742 (77.0)	9274 (80.3)	9042 (78.7)	4857 (74.9)	5903 (75.2)	6238 (75.5)
Female, n (%)	2210 (29.0)	2458 (28.5)	2638 (30.7)	2790 (28.7)	2858 (27.9)	2995 (27.0)	3062 (26.7)	2542 (22.1)	2604 (23.0)	2282 (19.7)	2446 (21.3)	1624 (25.1)	1944 (24.8)	2024 (24.5)
Age														
≤ 35y, n (%)	5993 (78.7)	6702 (77.6)	6698 (78.0)	7414 (76.2)	7812 (76.4)	8523 (76.7)	9237 (80.6)	9110 (79.1)	9055 (79.8)	9136 (79.1)	9071 (79.0)	4722 (72.9)	5966 (76.0)	6239 (75.5)
> 35y, n (%)	1626 (21.3)	1932 (22.4)	1884 (22.0)	2314 (23.8)	2417 (23.6)	2589 (23.3)	2220 (19.4)	2400 (20.9)	2291 (20.2)	2420 (20.9)	2417 (21.0)	1759 (27.1)	1881 (24.0)	2023 (24.5)
Blood donation history^a^														
None, n (%)	3791 (49.8)	4166 (48.3)	4581 (53.4)	5134 (52.8)	5615 (54.9)	5985 (53.9)	6308 (55.1)	6129 (53.2)	5945 (52.4)	5575 (48.2)	5718 (49.8)	1631 (25.2)	2469 (31.5)	1913 (23.2)
WB, n (%)	906 (11.9)	1331 (15.4)	1132 (13.2)	1519 (15.6)	1484 (14.5)	1875 (16.9)	1810 (15.8)	1831 (15.9)	1660 (14.6)	1665 (14.4)	1560 (13.6)	1352 (20.9)	1429 (18.2)	1446 (17.5)
PLT, n (%)	1793 (23.5)	1925 (22.3)	1755 (20.5)	1880 (19.3)	1899 (18.6)	1941 (17.5)	1971 (17.2)	2098 (18.2)	2153 (19.0)	2526 (21.9)	2373 (20.7)	1849 (28.5)	1874 (23.9)	2301 (27.9)
Both, n (%)	1129 (14.8)	1212 (14.0)	1114 (13.0)	1195 (12.3)	1231 (12.0)	1311 (11.8)	1368 (11.9)	1452 (12.6)	1588 (14.0)	1790 (15.5)	1837 (16.0)	1649 (25.4)	2075 (26.4)	2602 (31.5)
FRD in current interval														
Yes, n (%)	2194 (15.4)	3690 (24.1)	3654 (24.2)	4898 (28.9)	5865 (34.2)	6720 (36.1)	6633 (34.7)	7840 (40)	7435 (39.2)	8485 (41.9)	3677 (18.5)	0 (0.0)	0 (0.0)	0 (0.0)
No, n (%)	12,031 (84.6)	11,634 (75.9)	11,446 (75.8)	12,062 (71.1)	11,303 (65.8)	11,910 (63.9)	12,498 (65.3)	11,765 (60)	11,513 (60.8)	11,750 (58.1)	16,176 (81.5)	17,742 (100.0)	22,023 (100.0)	25,222 (100.0)

**Bold cross-sections** denote the ones after the ban on FRD

^a^“None” = no blood donation history; “WB” = whole blood donation history only; “PLT” = plateletpheresis donation history only; “Both” = both whole blood and plateletpheresis donations history

In either GZ or CD dataset, both average total number of donations and average total apheresis platelet units were significantly increased after the ban compared to those before the ban; in contrast, the change of average total number of donors from before to after the ban was the opposite (t = 2.42–5.56, *p* = 0.0325–0.0001) (Table 2, Additional file 3 and Additional file 4). These results indicated that there is a phenomenon of “fewer donors, more blood” after the FRD ban in plateletpheresis donation practice.

**Table 2:** Comparisons of total number of plateletpheresis donors, donations, and units before and after the ban

	Mean (SD)		t	***p***^**a**^
	Before the ban	After the ban		
**Overall GZ Set**				
Total #donors	10,297 (1442)	7530 (934)	3.10	0.0092
Total #donations	17,744 (2108)	21,662 (3753)	2.45	0.0308
Total units (U)	28,781 (3959)	38,425 (7417)	3.14	0.0085
**Overall CD Set**				
Total #donors	6170 (975)	4754 (317)	2.42	0.0325
Total #donations	9349 (1843)	12,469 (392)	2.83	0.0150
Total units (U)	12,803 (2250)	20,558 (1488)	5.56	0.0001

^a^Independent t-tests (two-tailed)

Additional file 5 demonstrated that both average apheresis platelet units per donor and average total number of plateletpheresis donations per donor after the ban were significantly higher than those before the ban for all covariates including gender, age, and blood donation history in both datasets (all *p*-values< 0.05). Further comparisons revealed that male donors or donors with plateletpheresis donation history had significantly larger increase (i.e., after-before mean difference) of both average apheresis platelet units per donor and average total number of plateletpheresis donations per donor compared to their peer groups (i.e., female donors or donors with other donation history) (all *p*-values< 0.05).

In addition, in terms of average apheresis platelet units per donor among family/replacement donors, voluntary donors before the ban, and voluntary donors after the ban, the family/replacement donors contributed the least, voluntary donors before the ban the second, and voluntary donors after the ban the most (ANOVA with post-hoc Tukey tests, all *p*-values< 0.0001) (Additional file 6).

### Model-based trend of average apheresis platelet units per donor over time in overall GZ set

**Fig. 1:**
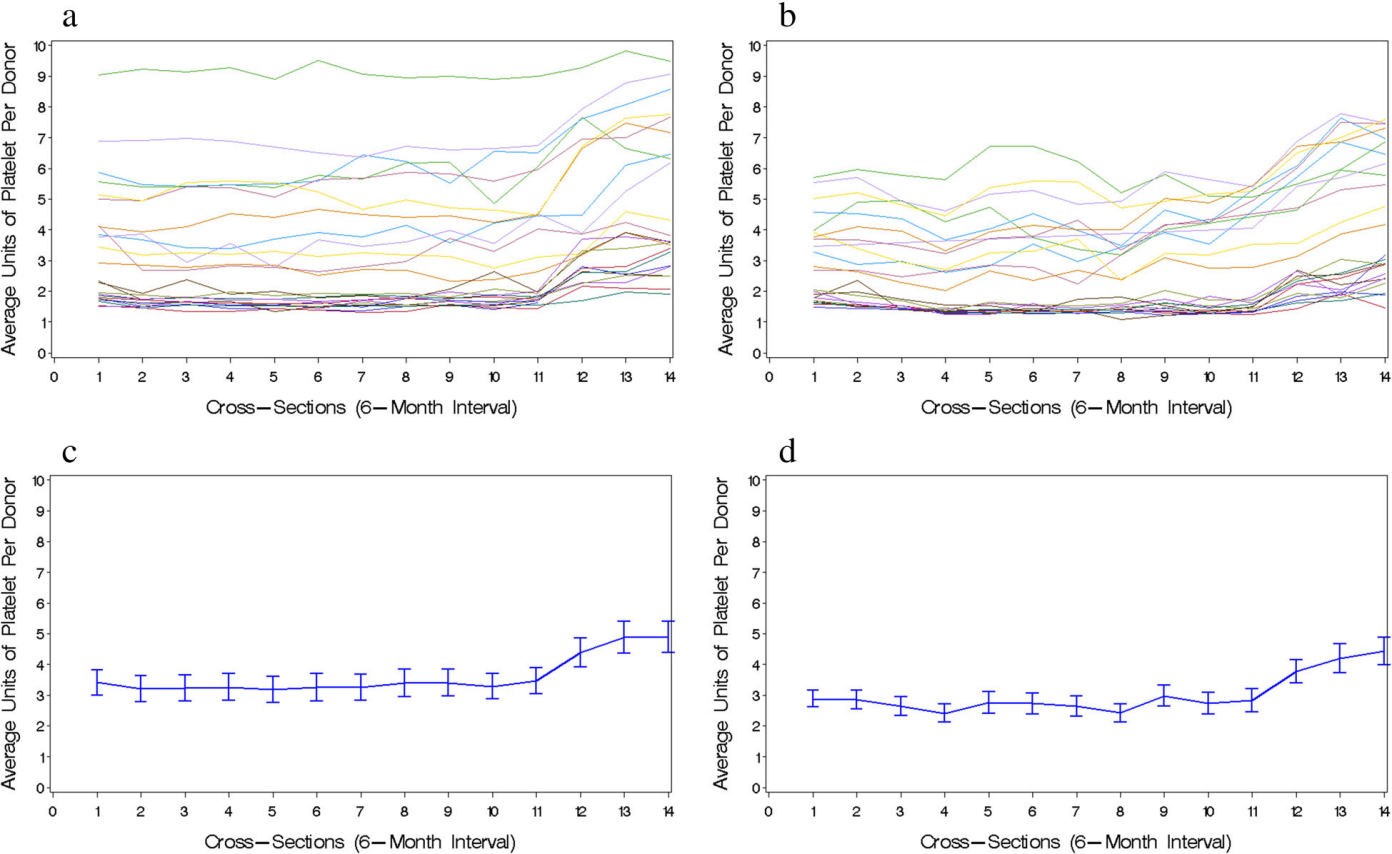
Visualization of the trend of outcome - average units of apheresis platelets per donor over time (i.e., cross-section) for overall GZ and CD pseudo-panel datasets. **a** and **b** visualize individual cohort group trajectories (outcome vs. time) with each line representing a trajectory of a cohort group who had common gender, birth year, and blood donation history for overall GZ and CD pseudo-panel datasets, respectively; **c** and **d** present the trend of overall mean outcome over time for overall GZ and CD pseudo-panel datasets, respectively

Additional file 7 summarized the characteristics of two independent pseudo-panel datasets (overall GZ set: *n* = 330 cells; overall CD set: *n* = 316 cells) with the number of plateletpheresis donors per cell ≥30. Figure 1a and b showed 24 individual trajectories of the average apheresis platelet units per donor representing 24 cohort groups with each who shared common gender, birth year, and blood donation history over 14 cross-sections in the overall GZ and CD pseudo-panel sets, respectively. Figure 1c and d visualized an overall mean profile of the outcome over time in the GZ and CD sets, respectively, indicating that there was a breakpoint at the 11th cross-section (just right before the ban) with a roughly horizontal line to the left of the breakpoint and significantly positive linear trend to the right.

**Fig. 2:**
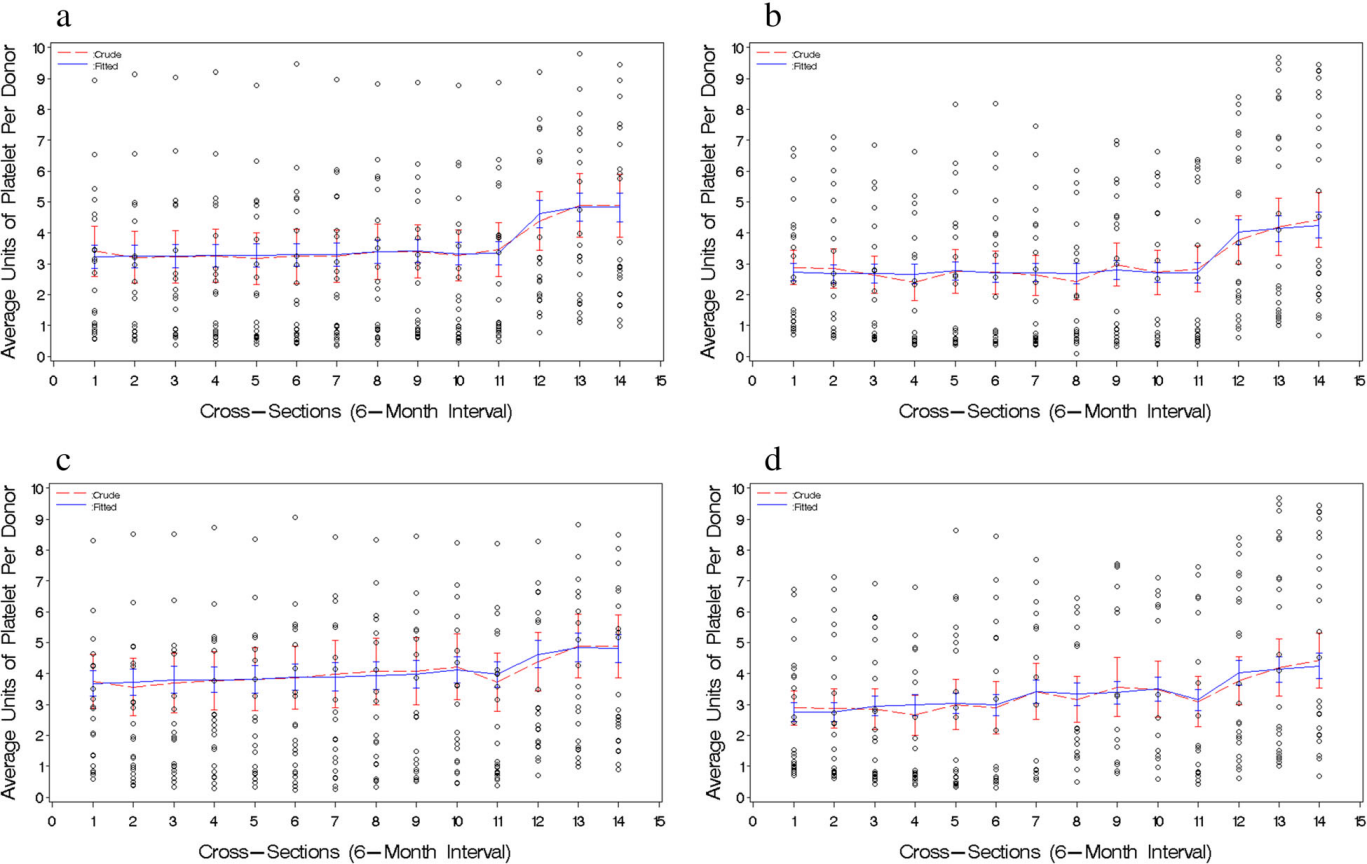
Visualization of the final two-piecewise linear mixed effects models from overall GZ / CD and voluntary GZ / CD pseudo-panel datasets. **a** and **b** visualize how close both the fitted and the crude trends of outcome mean over time are for overall GZ and CD pseudo-panel datasets, respectively. **c** and **d** visualize how close both the fitted and the crude trends of outcome mean over time are for voluntary GZ and CD pseudo-panel subsets, respectively. The dotted line represents the crude trend and the solid line the fitted trend. The vertical lines depict standard errors of the outcome means. The scattered circles are raw pseudo-panel data

Thus, to the overall GZ set, we fitted a two-piecewise linear mixed-effects model in which we specified intercept as the random term with an unstructured covariance-structure. The covariate birth year was excluded due to its non-significance. As shown in Table 3 and Fig. 2a, the final model presented a two-piecewise linear trend of average apheresis platelet units per donor over time (i.e., cross-section) with a horizontal line to the left of the breakpoint (β_timeBefore11_ = 0.0111, *p* = 0.0976) and a significantly positive linear trend to the right (β_timeAfter11_ = 0.0404, *p* < 0.0001). This result suggests that the average apheresis platelet units per donor were maintained at lower level and did not change with time before the ban, but started increasing linearly with time after the ban.

**Table 3:** Final models derived from two independent overall pseudo-panel datasets – overall GZ set (*n* = 330 cells) and overall CD set (*n* = 316 cells)

	Overall GZ Set^**a**^			Overall CD Set^**b**^		
	β	SE	***p***	β	SE	***p***
Intercept	0.8636	0.4643	0.0782	0.9637	0.2180	**0.0002**
timeBefore11	0.0111	0.0067	0.0976	0.0077	0.0084	0.3582
timeAfter11	0.0404	0.0093	**< 0.0001**	0.0441	0.0102	**< 0.0001**
Gender						
male vs. female	1.3370	0.4138	**0.0044**	0.7449	0.1909	**0.0009**
Blood donation history^c^						
WB vs. None	0.1790	0.5852	0.7630	0.1928	0.2687	0.4816
PLT vs. None	2.6243	0.5851	**0.0003**	2.1231	0.2690	**< 0.0001**
Both vs. None	3.9440	0.5851	**< 0.0001**	3.2293	0.2705	**< 0.0001**
timeAfter11 × gender						
time×male vs. time×female	0.0550	0.0072	**< 0.0001**	0.0325	0.0086	**0.0002**
time_After11_ × history						
time×WB vs. time×none	0.0331	0.0105	**0.0018**	0.0069	0.0120	0.5655
time×PLT vs. time×none^d^	0.0698	0.0104	**< 0.0001**	0.1373	0.0120	**< 0.0001**
time×Both vs. time×none^d^	0.0444	0.0104	**< 0.0001**	0.0507	0.0120	**< 0.0001**

^a^Model equation for Overall GZ set can be drew as: average apheresis platelet units per donor = 0.8630 + 0.0111*time_Before11_ + 0.0404*time_After11_ + 1.3370*(gender = male) + 0.0000*(gender = female) + 0.1790*(history = WB) + 2.6242*(history = PLT) + 3.9440*(history = Both) + 0.0000*(history = None) + 0.0550*time_After11_*(gender = male) + 0.0000*time_After11_*(gender = female) + 0.0331*time_After11_*(history = WB) + 0.0698*time_After11_*(history = PLT) + 0.0444*time_After11_*(history = Both) + 0.0000*time_After11_*(history = None)

^b^Model equation for Overall CD set can be drew as: average apheresis platelet units per donor = 0.9637 + 0.0077*time_Before11_ + 0.0441*time_After11_ + 0.7449*(gender = male) + 0.0000*(gender = female) + 0.1928*(history = WB) + 2.1231*(history = PLT) + 3.2293*(history = Both) + 0.0000*(history = None) + 0.0325*time_After11_*(gender = male) + 0.0000*time_After11_*(gender = female) + 0.0069*time_After11_*(history = WB) + 0.1373*time_After11_*(history = PLT) + 0.0507*time_After11_*(history = Both) + 0.0000*time_After11_*(history = None)

^c^“None” = no blood donation history; “WB” = whole blood donation history only; “PLT” = plateletpheresis donation history only; “Both” = both whole blood and plateletpheresis donations history

^d^The difference of the outcome change between time*PLT and time*Both was tested using Z = abs(β_2_– β_1_)/SQRT (SE_1_^2^ + SE_2_^2^), α = 0.05, one-tailed. For overall GZ set: Z = 1.727, *p* = 0.0421; for overall CD set: Z = 5.103, *p* < 0.0001

**Bold** values denote statistical significance

In the model, independent covariates measured whether baseline outcome differed by group. Table 3 demonstrated that on average at baseline, male donated 1.337 more apheresis platelet units per donor than female (β_male vs female_ = 1.3370, *p* = 0.0044), and the donated apheresis platelet units per donor was significantly higher in the donors who had platelet donation history than that in their peers who had no blood donation history (β_PLT vs None_ = 2.6243,*p* = 0.0003; β_Both vs None_ = 3.9440, *p* < 0.0001) whereas donations by the donors with whole blood donation history only were not significant (β_WB vs None_ = 0.1790, *p* = 0.7630). Due to the non-significance of the slope for the time_Before11_ term, we only considered the interactions between the time_After11_ and covariates in the model. After the model selection, a two-way interaction term gender×blood donation history and a three-way interaction term time_After11_ × gender×blood donation history were excluded due to their non-significance. The regression coefficient of the interaction term measured whether the outcome change differed by covariate-specific groups. As shown in Table 3, on average after the ban, the outcome change was significant in males than that in females (β_time × male vs time×female_ = 0.0550, *p* < 0.0001) and in donors with blood donation history than that in their peers without donation history (β_time × WB vs time×none_ = 0.0331, *p* = 0.0018; β_time × PLT vs time×none_ = 0.0698, *p* < 0.0001; and β_time × Both vs time×none_ = 0.0444,*p*< 0.0001). These results suggest that the contributions of gender and blood donation history to the model had heterogeneity for both the baseline outcome value and the outcome change after the ban.

The Pearson residual plots, either marginally for the consideration of fixed effects only or conditionally for the consideration of both fixed and random effects, for the final model indicated that the model’s assumptions – normality and heteroscedasticity were not significantly violated (Additional file 8). Five-fold cross-validation demonstrated that the over-fitting percentage of the final model for overall GZ set accounted for only 0.28% (Additional file 9), suggesting that there was no significant over-fitting issue for the final model.

### Replication of the model-based trend of average apheresis platelet units per donor over time in overall CD set

Next, we used an independent overall CD set to test the replication of the final model obtained from the overall GZ set. As shown in Table 3 and Fig. 2b**,** all parameters’ estimates in the final model for the overall CD set were similar to those for the overall GZ set, except for that the outcome change for the donors with whole blood donation history only was not significant and that the outcome values across all time-points appeared to be systematically reduced in the overall CD set, compared to those in the overall GZ set. The latter result was consistent with that from the individual-level data, i.e., the overall average apheresis platelet units per donor in Guangzhou (overall mean = 3.3 U, SD = 1.0) was higher than that in Chengdu (overall mean = 2.6 U, SD = 1.0) with a marginally non-significance (mean difference = 0.7 U, 95% CI:-0.04–1.5, *p* = 0.0644) (data not shown). The assumptions of normality and heteroscedasticity were not significantly violated (Additional file 8), and the over-fitting percentage of the final model for overall CD set was 2.52% (Additional file 9). These results suggest that the two-piecewise linear trend of the average apheresis platelet units per donor over time observed in overall GZ set can be replicated in an independent dataset – overall CD set with some minor variations.

### Effect of family/replacement platelet donations on model-based trend of the average apheresis platelet units per donor over 14 cross-sections in both GZ and CD sets

After removing the FRD across the first 11 cross-sections followed by re-grouping the data (Additional file 10), our modeling results demonstrated that although the average apheresis platelet units per donor still significantly increased over time after the ban (β_timeAfter11_ = 0.0588 and 0.0583 for voluntary GZ and CD subsets, respectively, all *p*-values < 0.0001), a positive linear trend of the outcome values over time before the ban also became significant (β_timeAfter11_ = 0.0400 and 0.0499 for voluntary GZ and CD subsets, respectively, all p-values < 0.0001) (Additional file 11, Fig. 2c and d). These results suggest that the FRD is a critical factor to influence the trend of the outcome values over time before the ban. The heterogeneity for group-specific baseline outcome and outcome change after the ban was still similar to that from the overall GZ and CD sets with some variations. Neither significant violations of the model’s assumptions (Additional file 12) nor over-fitting issue (over-fitting percentage was 4.64% for voluntary GZ subset and 0.65% for voluntary CD subset) (Additional file 13) were found for the final models.

## Discussion

In the present study, we observed that total number of donations and total apheresis platelet units after the ban were significantly higher than those before the ban whereas total number of plateletpheresis donors showed the opposite in both Guangzhou and Chengdu Blood Centers. These findings quantitatively confirmed an emerging phenomenon of “fewer donors, more blood (platelet units)” that we preliminarily observed in the field since the implement of the FRD ban in China under the circumstance of no substantial difference in the publicity and recruitment of blood donation between before and after the FRD ban in the two blood centers, which breaks the belief of “more donors, more blood (platelet units)” in the past plateletpheresis donation practice. It must be noted that we did not include whole blood donors/donations in our analyses due to that 1) the minimum interval between whole blood donations in China is 180 days (GB 18467–2011) and within a single cross-section (6-month interval) in our present study, the whole blood donors had no repeated whole blood donation(s); and 2) almost 100% whole blood had been donated by the VNRBDs during the 5 years prior to the FRD ban in China, and thus, the FRD ban would be expected to have no significant influence on the whole blood donations.

Furthermore, using a pseudo-panel data approach, we observed that the average apheresis platelet units per donor presented a horizontal line over time before the ban, and then followed a significantly positive linear trend over time after the ban from both GZ and CD datasets. To our knowledge, this study is the first to report such a two-piecewise linear trend of the average apheresis platelet units per donor over time before and after a nationwide FRD ban. Such a change of the donation trend might be related to a difference of the blood donors’ composition between before and after the ban. For apheresis platelet donations, before the FRD ban, the FRDs were dominated by the first-time blood donors, and they generally did not donate blood repeatedly. After the FRD ban, although the total number of blood donors had decreased, the proportion of the first-time blood donors was also decreased but more blood donors repeatedly donated. One possible reason for the plateletpheresis donors donating more blood and more often was that as the total number of blood donors was decreased, the average time for staff to communicate and serve with each blood donor was increased. Thus, the VNRBDs were more likely to experience a better blood donation service, which might increase the donors’ donations. We cannot exclude the possibility of other reasons that could enhance the loyalty and commitment of these donors. More rigorous studies are needed for further clarification.

Our modeling results also revealed that male donors and donors with plateletpheresis donation history had an increased baseline outcome and a significant outcome change over time after the ban. These findings are consistent with those from our individual-level data, i.e., both male donors and donors with plateletpheresis donation history had significantly larger increases of both average apheresis platelet units per donor and average total number of plateletpheresis donations per donor compared to their peer groups (i.e., female donors or donors with other donation history), which may be defined as improved plateletpheresis donation behavior. Therefore, a possible mechanism underlying the two-piecewise linear trend of the outcome over time before and after the ban could be the FRD ban-related improved plateletpheresis donation behavior. Evidence showed that the improved donation behavior can be related to the increased altruism [21, 22] and social responsibility of blood donors [21] whereas repeat donors had a higher return donation rate with altruistic reasons [22]. On the other hand, studies also demonstrated that male donors more frequently donated blood [22, 23] and women were less likely to donate blood [24, 25] probably due to the pregnancy- and/or lactation-based absence [24]. However, whether the ban can increase altruism and social responsibility of male donors and/or repeat donors is unknown. Another possible mechanism could be directly related to the implement of the FRD ban. Our findings indicated that compared to voluntary plateletpheresis donors, family/replacement donors donated significantly smaller volume of apheresis platelet, and therefore, removing family/replacement donors from the data led to the trend of outcome over time before the ban being changed from a horizontal line to a weak and positive linear line. Taken together, the two-piecewise linear trend of outcome over time may be an integrative result from two ban-related factors: the implement of the FRD ban and the improved donation behavior of male donors and/or donors with platelet donation history after the ban.

Systematic reduction of the outcome for the model as a whole in the samples from Chengdu Blood Center compared to that from Guangzhou Blood Center is consistent with our previous study, in which we reported that the average blood donation volume per resident was higher in Guangdong, whose capital city is Guangzhou, than that in Sichuan, whose capital city is Chengdu (3.06 U for Guangdong vs. 2.56 U for Sichuan) [26].

Our study has several strengths. The use of an independent external dataset (CD dataset) to replicate the findings from the GZ dataset significantly increased the external validity of our findings. The internal validity of our results was improved by the application of five-fold cross-validation. As mentioned above, our datasets presented a pseudo-panel structure, thus, the use of a pseudo-panel data approach maximized the reliability and validity of our analysis. Our study also has some limitations. Only three covariates were available for our modeling in both GZ and CD datasets, thus, we cannot completely rule out the possible confounding effects of the unmeasured covariates. However, by calculating the linear mixed- effects model’s R-square values (1-SSE/(SSE + SSR)) [27], about 80–89% of the variance for the outcome variable can be explained by the independent variables that are included in our final models in overall GZ and CD datasets, respectively (data not shown). The nationwide FRD ban in China was effective on April 1, 2018, thus, the number of the cross-sections available after the ban was relatively few and further continuously monitoring the trend of the outcome over time after the ban is needed. Our study was a cross-sectional design, thus, couldn’t figure out the causal relationship between the covariates and the outcome. Pseudo-panel data approach is an aggregation method, therefore, we lost some information and statistical power. We did not include whole blood donors/donations in our analyses due to the reasons discussed in the Methods, and thus, our findings are plateletpheresis-specific and cannot be generalized to whole blood donations.

## Conclusion

In conclusion, the significant increase of the average apheresis platelet units per donor over time after the FRD ban may be related to the implement of the FRD ban and the improved donation behavior of male donors and/or donors with platelet donation history after the ban. Our findings suggest that to further increase the plateletpheresis donations in China, a continuous implement of the FRD ban is encouraged and more rigorous blood donation motivations are needed for those donors who are female and who have no donation history. They may also potentially motivate the policymakers in other countries where the FRD for plateletpheresis donation is still legitimate to phase out their FRD strategy and ultimately achieve 100% voluntary plateletpheresis donation.

## Supplementary Information

**Additional file 1.** STROBE Statement—checklist of items that should be included in reports of observational studies.

**Additional file 2.** Demographics of individual plateletpheresis donors in CDBC.

**Additional file 3.** Total number of plateletpheresis donations/donors/units and average number of plateletpheresis donations/units in GZBC.

**Additional file 4.** Total number of plateletpheresis donations/donors/units and average number of plateletpheresis donations/units in CDBC.

**Additional file 5.** Comparisons of average plateletpheresis donation units per donor before and after the ban for individuals.

**Additional file 6.** Comparison of average plateletpheresis units per donor in both datasets.

**Additional file 7.** Characteristics of two independent pseudo-panel datasets (the number of plateletpheresis donors per cell ≥30).

**Additional file 8.** Examinations of the assumptions – normality and homoscedasticity of errors for the final mixed effects models derived from overall GZ (panels A and B) and CD (panels C and D) pseudo-panel datasets. Panels A and C present marginal Pearson residual plots, which are for the consideration of fixed effects only. Panels B and D visualize conditional Pearson residual plots, which are for the consideration of both fixed and random effects. The plot residuals vs. predicted outcome mean is used for examining the homoscedasticity assumption whereas the plots percent vs. residual and residual vs. quantiles are used for visualizing the normality assumption. The variable “pv” represents outcome, i.e., average units of platelet per donor.

**Additional file 9.** Five-fold cross-validation of the final models for overall pseudo-panel datasets.

**Additional file 10.** Characteristics of two independent pseudo-panel datasets (the number of plateletpheresis donors per cell ≥30).

**Additional file 11.** Final models derived from independent voluntary pseudo-panel datasets – voluntary GZ subset and voluntary CD subset.

**Additional file 12.** Examinations of the assumptions – normality and homoscedasticity of errors for the final mixed effects models derived from voluntary GZ (panels A and B) and CD (panels C and D) pseudo-panel datasets. Panels A and C present marginal Pearson residual plots, which are for the consideration of fixed effects only. Panels B and D visualize conditional Pearson residual plots, which are for the consideration of both fixed and random effects. The plot residuals vs. predicted outcome mean is used for examining the homoscedasticity assumption whereas the plots percent vs. residual and residual vs. quantiles are used for visualizing the normality assumption. The variable “pv” represents outcome, i.e., average units of platelet per donor.

**Additional file 13.** Five-fold cross-validation for the final models of voluntary pseudo-panel datasets.

## Data Availability

The datasets used and analyzed during the current study are available from the corresponding author on reasonable request.

## Abbreviations

FRD: Family/replacement blood donation
GZ: Guangzhou
GZBC: Guangzhou Blood Center
CD: Chengdu
CDBC: Chengdu Blood Center
GB18467–2011: The China national standard for the eligible donor selection criteria
WB: Whole Blood Donation Only
PLT: Apheresis Platelet Donation Only
Both: Both WB and PLT Donations
overall GZ set: Overall Guangzhou pseudo-panel set
overall CD set: Overall Chengdu pseudo-panel set
U: Units
RMSE: Root Mean Square Error
