# Supplementary material for: The apheresis platelet donation was increased after a nationwide ban on family/replacement donation in China
Source: BMC Public Health. 2021 Apr 29;21:819. doi: 10.1186/s12889-021-10819-4 (PMC8082857; doi:10.1186/s12889-021-10819-4)
Supplement: Supplementary file 2 — Additional file 2. Demographics of individual plateletpheresis donors in CDBC. [file 12889_2021_10819_MOESM2_ESM.pdf]

Additional file 2. Demographics of individual plateletpheresis donors in CDBC.

	2012/10-2013/3	2013/4-2013/9	2013/10-2014/3	2014/4-2014/9	2014/10-2015/3	2015/4-2015/9	2015/10-2016/3	2016/4-2016/9	2016/10-2017/3	2017/4-2017/9	2017/10-2018/3	**2018/4-2018/9**	**2018/10-2019/3**	**2019/4-2019/9**
Gender														
Male, n (%)	2 786 (66.7)	3 156 (64.8)	4 076 (74.4)	4 456 (74.1)	4 547 (72.3)	4 612 (73.4)	4 842 (73.2)	5 291 (72.1)	5 013 (72.1)	5 305 (75.5)	5 107 (75.2)	3 435 (67.2)	3 075 (66.5)	2 864 (63.3)
Female, n (%)	1 393 (33.3)	1 717 (35.2)	1 402 (25.6)	1 561 (25.9)	1 743 (27.7)	1 675 (26.6)	1 777 (26.8)	2 052 (27.9)	1 944 (27.9)	1 725 (24.5)	1 686 (24.8)	1 680 (32.8)	1 551 (33.5)	1 658 (36.7)
Age														
≤35y, n (%)	2 617 (62.6)	3 010 (61.8)	3 511 (64.1)	3 898 (64.8)	4 077 (64.8)	4 284 (68.1)	4 512 (68.2)	5 272 (71.8)	5 086 (73.1)	5 228 (74.4)	4 970 (73.2)	3 442 (67.3)	3 131 (67.7)	3 170 (70.1)
>35y, n (%)	1 562 (37.4)	1 863 (38.2)	1 967 (35.9)	2 119 (35.2)	2 213 (35.2)	2 003 (31.9)	2 107 (31.8)	2 071 (28.2)	1 871 (26.9)	1 802 (25.6)	1 823 (26.8)	1 673 (32.7)	1 495 (32.3)	1 352 (29.9)
Blood donation history[a]														
None, n (%)	2 483 (59.4)	3 005 (61.7)	3 798 (69.3)	4 271 (71.0)	4 398 (69.9)	4 200 (66.8)	4 569 (69.0)	5 051 (68.8)	4 480 (64.4)	4 332 (61.6)	4 006 (59.0)	1 847 (36.1)	1 592 (34.4)	1 250 (27.6)
WB, n (%)	468 (11.2)	621 (12.7)	592 (10.8)	651 (10.8)	739 (11.7)	891 (14.2)	780 (11.8)	879 (12)	889 (12.8)	856 (12.2)	959 (14.1)	984 (19.2)	664 (14.4)	666 (14.7)
PLT, n (%)	699 (16.7)	722 (14.8)	606 (11.1)	617 (10.3)	694 (11)	726 (11.5)	774 (11.7)	848 (11.5)	979 (14.1)	1 171 (16.7)	1 105 (16.3)	1 347 (26.3)	1 278 (27.6)	1 419 (31.4)
Both, n (%)	529 (12.7)	525 (10.8)	482 (8.8)	478 (7.9)	459 (7.3)	470 (7.5)	496 (7.5)	565 (7.7)	609 (8.8)	671 (9.5)	723 (10.6)	937 (18.3)	1 092 (23.6)	1 187 (26.2)
FRD donation in current interval														
Yes, n (%)	386 (5.8)	828 (11.2)	2 789 (37.1)	3 906 (47.8)	3 096 (35.1)	2 879 (31.8)	4 980 (52.1)	5 643 (52.6)	4 689 (40.9)	4 798 (40.9)	3 625 (30.9)	0 (0.0)	0 (0.0)	0 (0.0)
No, n (%)	6 281 (94.2)	6 571 (88.8)	4 719 (62.9)	4 259 (52.2)	5 718 (64.9)	6 185 (68.2)	4 576 (47.9)	5 092 (47.4)	6 788 (59.1)	6 925 (59.1)	8 106 (69.1)	12386 (100.0)	12125 (100.0)	12895 (100.0)

Bold cross-sections denote the ones after the ban on FRD.

[a]:"None"=no blood donation history; "WB"=whole blood donation history only; "PLT"=plateletpheresis donation history only; "Both"=both whole blood and plateletpheresis donations history.
