# Supplementary material for: The apheresis platelet donation was increased after a nationwide ban on family/replacement donation in China
Source: BMC Public Health. 2021 Apr 29;21:819. doi: 10.1186/s12889-021-10819-4 (PMC8082857; doi:10.1186/s12889-021-10819-4)
Supplement: Supplementary file 3 — Additional file 3. Total number of plateletpheresis donations/donors/units and average number of plateletpheresis donations/units in GZBC. [file 12889_2021_10819_MOESM3_ESM.pdf]

**Additional file 3. Total number of plateletpheresis donations/donors/units and average number of plateletpheresis donations/units in GZBC.**

| | 2012/10 -2013/3 | 2013/4 -2013/9 | 2013/10 -2014/3 | 2014/4 -2014/9 | 2014/10 -2015/3 | 2015/4 -2015/9 | 2015/10 -2016/3 | 2016/4 -2016/9 | 2016/10 -2017/3 | 2017/4 -2017/9 | 2017/10 -2018/3 | **2018/4 -2018/9** | **2018/10 -2019/3** | **2019/4 -2019/9** |
|---|---|---|---|---|---|---|---|---|---|---|---|---|---|---|
| Total #donations | 14 225 | 15 324 | 15 100 | 16 960 | 17 168 | 18 630 | 19 131 | 19 605 | 18 948 | 20 235 | 19 853 | 17 742 | 22 023 | 25 222 |
| Total units | 22 971.5 | 24 271.8 | 23 843.8 | 26 838.5 | 27 326.0 | 29 653.8 | 30 636 | 31 796.5 | 31 557.5 | 33 906.8 | 33 790.2 | 30 625 | 39 261 | 45 389 |
| Total #donors | 7 619 | 8 634 | 8 582 | 9 728 | 10 229 | 11 112 | 11 457 | 11 510 | 11 346 | 11 556 | 11 488 | 6 481 | 7 847 | 8 262 |
| Average total #donations per donor | 1.9 | 1.8 | 1.8 | 1.7 | 1.7 | 1.7 | 1.7 | 1.7 | 1.7 | 1.8 | 1.7 | 2.7 | 2.8 | 3.1 |
| Average total units per donor | 3.0 | 2.8 | 2.8 | 2.8 | 2.7 | 2.7 | 2.7 | 2.8 | 2.8 | 2.9 | 2.9 | 4.7 | 5.0 | 5.5 |
| Gender | | | | | | | | | | | | | | |
| Average #donations per male | 2.0 | 1.9 | 1.9 | 1.9 | 1.8 | 1.8 | 1.8 | 1.8 | 1.8 | 1.9 | 1.8 | 3.1 | 3.1 | 3.4 |
| Average #donations per female | 1.5 | 1.4 | 1.4 | 1.4 | 1.3 | 1.3 | 1.3 | 1.3 | 1.3 | 1.4 | 1.4 | 1.8 | 2.0 | 2.0 |
| Average units per male | 3.3 | 3.1 | 3.1 | 3.1 | 3.0 | 3.0 | 3.0 | 3.0 | 3.0 | 3.2 | 3.2 | 5.4 | 5.6 | 6.2 |
| Average units per female | 2.2 | 2.0 | 2.0 | 2.0 | 1.9 | 1.9 | 1.9 | 2.0 | 2.0 | 2.0 | 2.1 | 2.8 | 3.2 | 3.3 |
| Age | | | | | | | | | | | | | | |
| Average #donations per donor (≤35) | 1.7 | 1.6 | 1.6 | 1.6 | 1.6 | 1.6 | 1.5 | 1.6 | 1.5 | 1.6 | 1.6 | 2.5 | 2.6 | 2.8 |
| Average #donations per donor (>35) | 2.3 | 2.2 | 2.2 | 2.1 | 2.0 | 2.0 | 2.2 | 2.2 | 2.2 | 2.3 | 2.3 | 3.3 | 3.5 | 3.7 |
| Average units per donor (≤35) | 2.8 | 2.6 | 2.5 | 2.6 | 2.5 | 2.5 | 2.4 | 2.5 | 2.5 | 2.7 | 2.6 | 4.3 | 4.5 | 5.1 |
| Average units per donor (>35) | 3.9 | 3.6 | 3.7 | 3.4 | 3.3 | 3.3 | 3.6 | 3.7 | 3.9 | 3.9 | 4.1 | 5.9 | 6.4 | 6.8 |
| FRD donation status | | | | | | | | | | | | | | |
| Average #donations per voluntary | 2.1 | 2.2 | 2.2 | 2.3 | 2.3 | 2.3 | 2.2 | 2.6 | 2.4 | 2.7 | 1.9 | 2.7 | 2.8 | 3.1 |
| Average #donations per FRD | 1.05 | 1.07 | 1.05 | 1.06 | 1.07 | 1.07 | 1.06 | 1.08 | 1.08 | 1.12 | 1.06 | NA | NA | NA |
| Average units per voluntary | 3.5 | 3.6 | 3.6 | 3.8 | 3.8 | 3.9 | 3.8 | 4.3 | 4.1 | 4.6 | 3.3 | 4.7 | 5.0 | 5.5 |
| Average units per FRD | 1.6 | 1.6 | 1.5 | 1.5 | 1.5 | 1.6 | 1.6 | 1.6 | 1.7 | 1.8 | 1.8 | NA | NA | NA |
| Blood donation history[a] | | | | | | | | | | | | | | |
| Average #donations per WB | 1.3 | 1.2 | 1.2 | 1.2 | 1.2 | 1.2 | 1.2 | 1.2 | 1.2 | 1.3 | 1.2 | 1.9 | 1.9 | 1.9 |
| Average #donations per PLT | 2.5 | 2.5 | 2.6 | 2.6 | 2.6 | 2.6 | 2.6 | 2.6 | 2.5 | 2.5 | 2.6 | 3.4 | 3.7 | 3.7 |
| Average #donations per Both | 3.4 | 3.4 | 3.4 | 3.5 | 3.4 | 3.4 | 3.2 | 3.3 | 3.1 | 3.0 | 2.9 | 3.8 | 4.1 | 4.1 |
| Average #donations per None | 1.2 | 1.2 | 1.2 | 1.2 | 1.1 | 1.2 | 1.2 | 1.2 | 1.1 | 1.2 | 1.1 | 1.6 | 1.5 | 1.8 |
| Average units per WB | 1.9 | 1.8 | 1.8 | 1.8 | 1.8 | 1.7 | 1.8 | 1.8 | 1.8 | 2.0 | 1.9 | 3.0 | 3.2 | 3.2 |
| Average units per PLT | 4.3 | 4.1 | 4.2 | 4.4 | 4.3 | 4.5 | 4.5 | 4.5 | 4.4 | 4.4 | 4.7 | 6.2 | 6.9 | 6.7 |
| Average units per Both | 6.1 | 6.0 | 6.2 | 6.3 | 6.1 | 6.0 | 5.6 | 5.8 | 5.6 | 5.4 | 5.4 | 6.8 | 7.6 | 7.6 |
| Average units per None | 1.8 | 1.6 | 1.7 | 1.6 | 1.6 | 1.6 | 1.7 | 1.7 | 1.7 | 1.8 | 1.7 | 2.4 | 2.5 | 2.9 |

**Bold cross-sections** denote the ones after the ban on FRD.

[a]"None"=no blood donation history; "WB"=whole blood donation history only; "PLT"=plateletpheresis donation history only; "Both"=both whole blood and plateletpheresis donations history.
