# Supplementary material for: The apheresis platelet donation was increased after a nationwide ban on family/replacement donation in China
Source: BMC Public Health. 2021 Apr 29;21:819. doi: 10.1186/s12889-021-10819-4 (PMC8082857; doi:10.1186/s12889-021-10819-4)
Supplement: Supplementary file 4 — Additional file 4. Total number of plateletpheresis donations/donors/units and average number of plateletpheresis donations/units in CDBC. [file 12889_2021_10819_MOESM4_ESM.pdf]

Additional file 4. Total number of plateletpheresis donations/donors/units and average number of plateletpheresis donations/units in CDBC.

	2012/10 -2013/3	2013/4 -2013/9	2013/10 -2014/3	2014/4 -2014/9	2014/10 -2015/3	2015/4 -2015/9	2015/10 -2016/3	2016/4 -2016/9	2016/10 -2017/3	2017/4 -2017/9	2017/10 -2018/3	**2018/4 -2018/9**	**2018/10 -2019/3**	**2019/4 -2019/9**
Total #donations	6 667	7 399	7 508	8 165	8 814	9 064	9 556	10 735	11 477	11 723	11 731	12 386	12 125	12 895
Total units	9 948.0	10 933.0	10 905.0	10 662.0	12 020.0	12 232.8	12 787.0	13 671.0	15 633.0	15 588.0	16 454.0	19 335.6	20 124.5	22 215.0
Total #donors	4 179	4 873	5 478	6 017	6 290	6 287	6 619	7 343	6 957	7 030	6 793	5 115	4 626	4 522
Average #donations per donor	1.6	1.5	1.4	1.4	1.4	1.4	1.4	1.5	1.6	1.7	1.7	2.4	2.6	2.9
Average units per donor	2.4	2.2	2.0	1.8	1.9	1.9	1.9	1.9	2.2	2.2	2.4	3.8	4.4	4.9
Gender														
Average #donations per male	2.0	1.9	1.9	1.9	1.8	1.8	1.8	1.8	1.8	1.9	1.8	3.1	3.1	3.4
Average #donations per female	1.5	1.4	1.4	1.4	1.3	1.3	1.3	1.3	1.3	1.4	1.4	1.8	2.0	2.0
Average units per male	3.3	3.1	3.1	3.1	3.0	3.0	3.0	3.0	3.0	3.2	3.2	5.4	5.6	6.2
Average units per female	2.2	2.0	2.0	2.0	1.9	1.9	1.9	2.0	2.0	2.0	2.1	2.8	3.2	3.3
Age														
Average #donations per donor (≤35)	1.5	1.4	1.3	1.3	1.3	1.4	1.4	1.4	1.6	1.6	1.7	2.4	2.6	2.8
Average #donations per donor (>35)	1.7	1.6	1.5	1.5	1.5	1.6	1.5	1.5	1.7	1.7	1.8	2.4	2.7	3.0
Average units per donor (≤35)	2.3	2.1	1.9	1.7	1.8	1.9	1.9	1.8	2.2	2.2	2.4	3.8	4.3	4.8
Average units per donor (>35)	2.5	2.4	2.2	1.9	2.1	2.1	2.0	1.9	2.3	2.3	2.5	3.6	4.5	5.1
FRD donation status														
Average #donations per voluntary	1.6	1.6	1.6	1.7	1.6	1.7	2.1	2.1	2.3	2.5	2.3	2.4	2.6	2.9
Average #donations per FRD	1.01	1.00	1.02	1.06	1.08	1.07	1.07	1.10	1.12	1.07	1.05	NA	NA	NA
Average units per voluntary	2.5	2.4	2.5	2.4	2.3	2.3	3.2	3.0	3.3	3.6	3.4	3.8	4.4	4.9
Average units per FRD	1.3	1.3	1.4	1.3	1.3	1.3	1.3	1.3	1.4	1.3	1.3	NA	NA	NA
Blood donation history[a]														
Average #donations per WB	1.4	1.3	1.1	1.2	1.2	1.2	1.2	1.3	1.4	1.3	1.3	1.7	1.6	1.7
Average #donations per PLT	2.2	2.2	2.1	2.1	2.3	2.5	2.5	2.5	2.9	2.8	3.1	3.5	3.6	3.7
Average #donations per Both	3.0	3.2	3.0	3.0	3.4	3.5	3.3	3.0	3.3	3.3	3.2	3.6	3.7	3.8
Average #donations per None	1.1	1.1	1.1	1.1	1.1	1.1	1.1	1.2	1.2	1.2	1.2	1.5	1.5	1.6
Average units per WB	1.9	1.8	1.6	1.4	1.5	1.5	1.5	1.5	1.8	1.6	1.6	2.4	2.4	2.6
Average units per PLT	3.6	3.6	3.5	3.1	3.6	3.8	3.7	3.5	4.5	4.3	4.9	5.8	6.4	6.6
Average units per Both	4.9	5.2	4.8	4.5	5.3	5.3	5.2	4.3	4.9	4.8	5.0	5.8	6.3	6.7
Average units per None	1.6	1.5	1.4	1.3	1.4	1.3	1.4	1.4	1.5	1.4	1.5	2.0	2.2	2.5

Bold cross-sections denote the ones after the ban on family/replacement donation.

[a]"None"=no blood donation history; "WB"=whole blood donation history only; "PLT"=plateletpheresis donation history only; "Both"=both whole blood and plateletpheresis donations history.
