# Supplementary material for: The apheresis platelet donation was increased after a nationwide ban on family/replacement donation in China
Source: BMC Public Health. 2021 Apr 29;21:819. doi: 10.1186/s12889-021-10819-4 (PMC8082857; doi:10.1186/s12889-021-10819-4)
Supplement: Supplementary file 5 — Additional file 5. Comparisons of average plateletpheresis donation units per donor before and after the ban for individuals. [file 12889_2021_10819_MOESM5_ESM.pdf]

Additional file 5. Comparisons of average plateletpheresis donation units per donor before and after the ban for individuals

	Average Plateletpheresis Donation Units Per Donor					Average Plateletpheresis Donation Units Per Donor				
	**Before Ban Mean (SD), n**	**After Ban Mean (SD), n**	**Mean Difference (SE)**	**t**	**p**	**Before Ban Mean (SD), n**	**After Ban Mean (SD), n**	**Mean Difference (SE)**	**t**	**p**
**Overall GZ set**										
Total	1.7(0.1),11	2.9(0.2),3	1.1(0.1)	9.19	0.0096	2.8(0.1),11	5.1(0.4),3	2.3(0.1)	9.60	0.0097
Gender										
male	1.9(0.1),11	3.2(0.2),3	**1.3(0.1)**	13.17	0.0041	3.1(0.1),11	5.7(0.4),3	**2.6(0.2)**	10.90	0.0074
female	1.4(0.1),11	1.9(0.1),3	0.6(0.1)	11.28	<0.0001	2.0(0.1),11	3.1(0.3),3	1.1(0.1)	7.09	0.0164
Age										
≤35 years	1.6(0.1),11	2.6(0.2),3	1.0(0.1)	11.62	0.0057	2.6(0.1),11	4.6(0.4),3	2.1(0.2)	8.53	0.0120
>35 years	2.2(0.1),11	3.5(0.2),3	1.3(0.1)	10.99	0.0047	3.7(0.3),11	6.4(0.5),3	2.7(0.3)	9.89	0.0054
Blood donation history[a]										
WB	1.2(<0.1),11	1.9(<0.1),3	0.7(<0.1)	55.9	<0.0001	1.8(0.1),11	3.1(0.1),3	1.3(0.1)	18.46	0.0009
PLT	2.6(0.1),11	3.6(0.2),3	**1.0(0.1)**	10.25	0.0080	4.4(0.2),11	6.6(0.4),3	**2.2(0.2)**	10.32	0.0063
Both	3.3(0.2),11	4.0(0.2),3	0.7(0.1)	6.27	0.0049	5.9(0.3),11	7.3(0.5),3	1.5(0.3)	5.18	0.0206
None	1.2(<0.1),11	1.6(0.2),3	0.5(0.1)	5.16	0.0320	1.7(0.1),11	2.6(0.3),3	0.9(0.2)	5.95	0.0245
FRD:										
FRD										
voluntary	2.3(0.2),11	2.9(0.2),3	0.6(0.1)	4.19	0.0198	3.8(0.4),11	5.1(0.4),3	1.2(0.3)	4.72	0.0180
**Overall CD set**										
Total	1.5(0.1),11	2.6(0.3),3	1.1(0.1)	11.39	<0.0001	2.1(0.2),11	4.4(0.6),3	2.3(0.2)	7.07	0.0156
Gender:										
male	1.6(0.2),11	3.0(0.3),3	**1.4(0.2)**	12.51	<0.0001	2.2(0.2),11	5.1(0.7),3	**2.8(0.4)**	6.91	0.0171
female	1.3(0.1),11	2.0(0.1),3	0.6(0.1)	10.32	<0.0001	1.8(0.2),11	3.0(0.5),3	1.2(0.2)	4.56	0.0385
Age										
≤35 years	1.4(0.1),11	2.6(0.2),3	1.2(0.1)	9.42	0.0049	2.0(0.2),11	4.3(0.5),3	2.3(0.3)	7.68	0.0119
>35 years	1.6(0.1),11	2.7(0.3),3	1.1(0.2)	6.24	0.0207	2.2(0.2),11	4.4(0.8),3	2.2(0.4)	4.99	0.0345
Blood donation history[a]										
WB	1.3(0.1),11	1.7(0.1),3	0.4(<0.1)	9.28	0.0002	1.6(0.2),11	2.5(0.1),3	0.9(0.1)	10.47	0.0003
PLT	2.5(0.3),11	3.6(0.1),3	**1.1(0.1)**	9.63	<0.0001	3.8(0.5),11	6.3(0.4),3	**2.4(0.3)**	8.48	0.0011
Both	3.2(0.2),11	3.7(0.1),3	0.5(0.1)	6.33	0.0007	4.9(0.3),11	6.3(0.5),3	1.3(0.3)	4.82	0.0237
None	1.1(0.1),11	1.5(0.1),3	0.4(<0.1)	10.83	0.0020	1.4(0.1),11	2.2(0.3),3	0.8(0.1)	5.45	0.0275
FRD status										
FRD	NA	NA	NA	NA	NA	NA	NA	NA	NA	NA
voluntary	1.9(0.3),11	2.6(0.3),3	0.7(0.2)	4.00	0.0136	2.8(0.5),11	4.4(0.6),3	1.6(0.4)	4.43	0.0221

Bold font indicates statistical significance for the indicated group vs. other group(s) within the same variable at the α=0.05 level. Z-test was used to compare two mean differences: Z=(mean difference1-mean difference2)/sqrt(se12+se22), two-tailed.

[a]"None"=no blood donation history; "WB"=whole blood donation history only; "PLT"=plateletpheresis donation history only; "Both"=both whole blood and plateletpheresis donations history.
