# Supplementary material for: The apheresis platelet donation was increased after a nationwide ban on family/replacement donation in China
Source: BMC Public Health. 2021 Apr 29;21:819. doi: 10.1186/s12889-021-10819-4 (PMC8082857; doi:10.1186/s12889-021-10819-4)
Supplement: Supplementary file 6 — Additional file 6. Comparison of average plateletpheresis units per donor in both datasets. [file 12889_2021_10819_MOESM6_ESM.pdf]

Additional file 6. Comparison of average plateletpheresis units per donor in both datasets.

	Mean Difference	95% CI of Mean	p
**Overall GZ Set**			
Voluntary Before vs. Voluntary After	1.2	0.7~1.7	<0.0001
Voluntary Before vs. FRD	2.2	1.9~2.6	<0.0001
Voluntary After vs. FRD	3.4	3.0~3.9	<0.0001
**Overall CD Set**			
Voluntary Before vs. Voluntary After	1.6	0.9~2.2	<0.0001
Voluntary Before vs. FRD	1.5	1.1~1.9	<0.0001
Voluntary After vs. FRD	3.0	2.4~3.7	<0.0001
**Overall GZ vs Overall CD**	0.7	-0.04-1.5	0.0644
