# Supplementary material for: The apheresis platelet donation was increased after a nationwide ban on family/replacement donation in China
Source: BMC Public Health. 2021 Apr 29;21:819. doi: 10.1186/s12889-021-10819-4 (PMC8082857; doi:10.1186/s12889-021-10819-4)
Supplement: Supplementary file 7 — Additional file 7. Characteristics of two independent pseudo-panel datasets (the number of plateletpheresis donors per cell ≥30). [file 12889_2021_10819_MOESM7_ESM.pdf]

Additional file 7. Characteristics of two independent pseudo-panel datasets (the number of plateletpheresis donors per cell ≥30)

Gender	Birth year	Blood donation history[a]				Total
		None<br>Number of cross-sections<br>(Min, Max)[b]	WB<br>Number of cross-sections<br>(Min, Max)	PLT<br>Number of cross-sections<br>(Min, Max)	Both<br>Number of cross-sections<br>(Min, Max)	
**Overall GZ Set**						
Male	1952-1974	14(63,489)	14(79,140)	14(156,234)	14(220,338)	56
Male	1975-1984	14(114,1051)	14(158,352)	14(312,435)	14(281,460)	56
Male	1985-2001	14(897,3528)	14(329,947)	14(737,1478)	14(356,1226)	56
Female	1952-1974	11(79,237)	11(30,55)	14(51,80)	14(42,77)	50
Female	1975-1984	14(55,425)	14(41,102)	14(53,87)	14(36,63)	56
Female	1985-2001	14(409,1396)	14(191,408)	14(246,357)	14(149,443)	56
Total		81	81	84	84	330
**Overall CD Set**						
Male	1952-1974	14(51,648)	14(44,173)	14(73,115)	14(91,174)	56
Male	1975-1984	14(79,807)	14(47,144)	14(98,151)	14(89,154)	56
Male	1985-2001	14(585,2481)	14(148,440)	14(207,851)	14(132,475)	56
Female	1952-1974	14(79,318)	11(31,160)	12(32,71)	12(30,125)	49
Female	1975-1984	14(57,279)	14(36,70)	10(32,55)	5(30,67)	43
Female	1985-2001	14(398,975)	14(75,230)	14(76,286)	14(48,201)	56
Total		84	81	78	73	316

[a]"None"=no blood donation history; "WB"=whole blood donation history only; "PLT"=plateletpheresis donation history only; "Both"=both whole blood and plateletpheresis donations history.

[b]Values in bracket are the minimum and maximum number of individual platelet donors in the cells across all involved cross-sections.
