# Supplementary material for: The apheresis platelet donation was increased after a nationwide ban on family/replacement donation in China
Source: BMC Public Health. 2021 Apr 29;21:819. doi: 10.1186/s12889-021-10819-4 (PMC8082857; doi:10.1186/s12889-021-10819-4)
Supplement: Supplementary file 9 — Additional file 9. Five-fold cross-validation of the final models for overall pseudo-panel datasets. [file 12889_2021_10819_MOESM9_ESM.pdf]

Additional file 9. Five-fold cross-validation of the final models for overall pseudo-panel datasets

	Overall GZ Set	Overall CD Set
RMSE1	1.0109	0.6282
RMSE2	0.8635	0.5398
RMSE3	1.0305	0.6885
RMSE4	0.9045	0.5328
RMSE5	1.0134	0.5433
$AverageRMSE_{(cross\text{-}validation)}$ [a]	0.9669	0.5898
$RMSE_{(full\ sample)}$	0.9642	0.5753
ΔRMSE [b]	0.0027	0.0145
% change in RMSE [c]	0.2800	2.5204

[a] $AverageRMSE_{(cross\text{-}validation)} = SQRT((RMSE_1^2 + RMSE_2^2 + \ldots + RMSE_5^2)/5)$

[b] $\Delta RMSE = Average\ RMSE_{(cross\text{-}validation)} - RMSE_{(full\ sample)}$

[c] % change in RMSE = $\Delta RMSE \times 100 / RMSE_{(full\ sample)}$
