# Supplementary material for: The apheresis platelet donation was increased after a nationwide ban on family/replacement donation in China
Source: BMC Public Health. 2021 Apr 29;21:819. doi: 10.1186/s12889-021-10819-4 (PMC8082857; doi:10.1186/s12889-021-10819-4)
Supplement: Supplementary file 10 — Additional file 10. Characteristics of two independent pseudo-panel datasets (the number of plateletpheresis donors per cell ≥30). [file 12889_2021_10819_MOESM10_ESM.pdf]

Additional file 10. Characteristics of two independent pseudo-panel datasets (the number of plateletpheresis donors per cell ≥ 30)

Gender	Birth year	Blood donation history[a]				Total
		None	WB	PLT	Both	
		Number of cross-sections (Min, Max)[b]	Number of cross-sections (Min, Max)	Number of cross-sections (Min, Max)	Number of cross-sections (Min, Max)	
**Voluntary GZ Subset**						
Male	1952-1974	14(63,186)	9(33,111)	14(138,205)	14(211,338)	51
Male	1975-1984	14(114,460)	14(42,209)	14(265,353)	14(246,460)	56
Male	1985-2001	14(625,2384)	14(135,719)	14(681,1 409)	14(324,1 226)	56
Female	1952-1974	10(32,98)	2(31,36)	14(48,71)	14(38,77)	40
Female	1975-1984	14(53,174)	4(37,52)	14(35,66)	9(30,63)	41
Female	1985-2001	14(260,768)	14(90,408)	14(213,328)	14(125,443)	56
Total		80	57	84	79	300
**Voluntary CD Subset**						
Male	1952-1974	14(37,340)	12(31,173)	14(63,110)	14(82,174)	54
Male	1975-1984	14(65,407)	14(32,102)	14(78,137)	14(78,154)	56
Male	1985-2001	14(432,1 037)	14(122,287)	14(177,851)	14(123,475)	56
Female	1952-1974	10(49,218)	6(44,160)	8(30,68)	10(33,125)	34
Female	1975-1984	10(49,195)	7(31,70)	6(34,55)	5(30,67)	28
Female	1985-2001	14(209,540)	14(63,225)	14(64,286)	14(43,201)	56
Total		76	67	70	71	284

[a]"None"=no blood donation history; "WB"=whole blood donation history only; "PLT"=plateletpheresis donation history only; "Both"=both whole blood and plateletpheresis donations history.

[b]Values in bracket are the minimum and maximum number of individual donors in the cells across all involved cross-sections.
