# Supplementary material for: The apheresis platelet donation was increased after a nationwide ban on family/replacement donation in China
Source: BMC Public Health. 2021 Apr 29;21:819. doi: 10.1186/s12889-021-10819-4 (PMC8082857; doi:10.1186/s12889-021-10819-4)
Supplement: Supplementary file 11 — Additional file 11. Final models derived from independent voluntary pseudo-panel datasets – voluntary GZ subset and voluntary CD subset. [file 12889_2021_10819_MOESM11_ESM.pdf]

Additional file 11. Final models derived from independent voluntary pseudo-panel datasets – voluntary GZ subset and voluntary CD subset

	**Voluntary GZ Subset[a]**			**Voluntary CD Subset[b]**		
	β	SE	p	β	SE	p
Intercept	0.6304	0.4531	0.1797	0.7783	0.2395	**0.0037**
timeBefore11	0.0400	0.0085	**<0.0001**	0.0499	0.0099	**<0.0001**
timeAfter11	0.0588	0.0115	**<0.0001**	0.0583	0.0114	**<0.0001**
Gender						
male vs. female	1.6519	0.4042	**0.0006**	0.9285	0.2110	**0.0003**
Blood donation history[c]						
WB vs. none	0.5769	0.5745	0.3274	0.2575	0.2971	0.3963
PLT vs. none	3.0199	0.5689	**<0.0001**	2.3244	0.2965	**<0.0001**
Both vs. none	4.3436	0.5692	**<0.0001**	3.2595	0.2966	**<0.0001**
timeAfter11×gender						
time×male vs. time×female	0.0307	0.0092	**0.0009**	0.0185	0.0098	0.0610
timeAfter11×history						
time×WB vs. time×none	0.0019	0.0137	0.8880	0.0019	0.0137	0.8883
time×PLT vs. time×none[d]	0.0390	0.0125	**0.0021**	0.1219	0.0136	**<0.0001**
time×Both vs. time×none[d]	0.0133	0.0126	0.2928	0.0484	0.0136	**0.0005**

[a]Model equation for voluntary GZ subset can be drew as: average platelet units per donor=0.6304 + 0.0400*timeBefore11 + 0.0588*timeAfter11 + 1.6519*(gender=male) + 0.0000*(gender=female) + 0.5769*(history=WB) + 3.0199*(history=PLT) + 4.3436*(history=Both) + 0.0000*(history=None) + 0.0307*timeAfter11*(gender=male) +0.0000*timeAfter11*(gender=female)+ 0.0019*timeAfter11*(history=WB) + 0.0390*timeAfter11*(history=PLT)+ 0.0133*timeAfter11*(history=Both) + 0.0000*timeAfter11*(history=None).

[b]Model equation for voluntary CD subset can be drew as: average platelet units per donor=0.7783 + 0.0499*timeBefore11 + 0.0583*timeAfter11 + 0.9285*(gender=male) + 0.0000*(gender=female) + 0.2575*(history=WB) + 2.3244*(history=PLT) + 3.2595*(history=Both) + 0.0000*(history=None) + 0.0185*timeAfter11*(gender=male) + 0.0000*timeAfter11*(gender=female)+ 0.0019*timeAfter11*(history=WB) + 0.1219*timeAfter11*(history=PLT) + 0.0484*timeAfter11*(history=Both) + 0.0000*timeAfter11*(history=None).

[c]Values in bracket are the minimum and maximum number of individual donors in the cells across all involved cross-sections.

[d]The difference of the outcome change between time*PLT and time*Both was tested using $Z=abs(\beta_2 - \beta_1)/SQRT(SE_1^2 + SE_2^2)$, $\alpha=0.05$, one-tailed. For voluntary CD subset: Z=3.821, p<0.0001.

Bold values denote statistical significance.
