# Supplementary material for: The apheresis platelet donation was increased after a nationwide ban on family/replacement donation in China
Source: BMC Public Health. 2021 Apr 29;21:819. doi: 10.1186/s12889-021-10819-4 (PMC8082857; doi:10.1186/s12889-021-10819-4)

Additional file 12. Examinations of the assumptions – normality and homoscedasticity of errors for the final mixed effects models derived from voluntary GZ (panels A and B) and CD (panels C and D) pseudo-panel datasets.

Panels A and C present marginal Pearson residual plots, which are for the consideration of fixed effects only. Panels B and D visualize conditional Pearson residual plots, which are for the consideration of both fixed and random effects. The plot residuals vs. predicted outcome mean is used for examining the homoscedasticity assumption whereas the plots percent vs. residual and residual vs. quantiles are used for visualizing the normality assumption. The variable "pv" represents outcome, i.e., average units of platelet per donor.

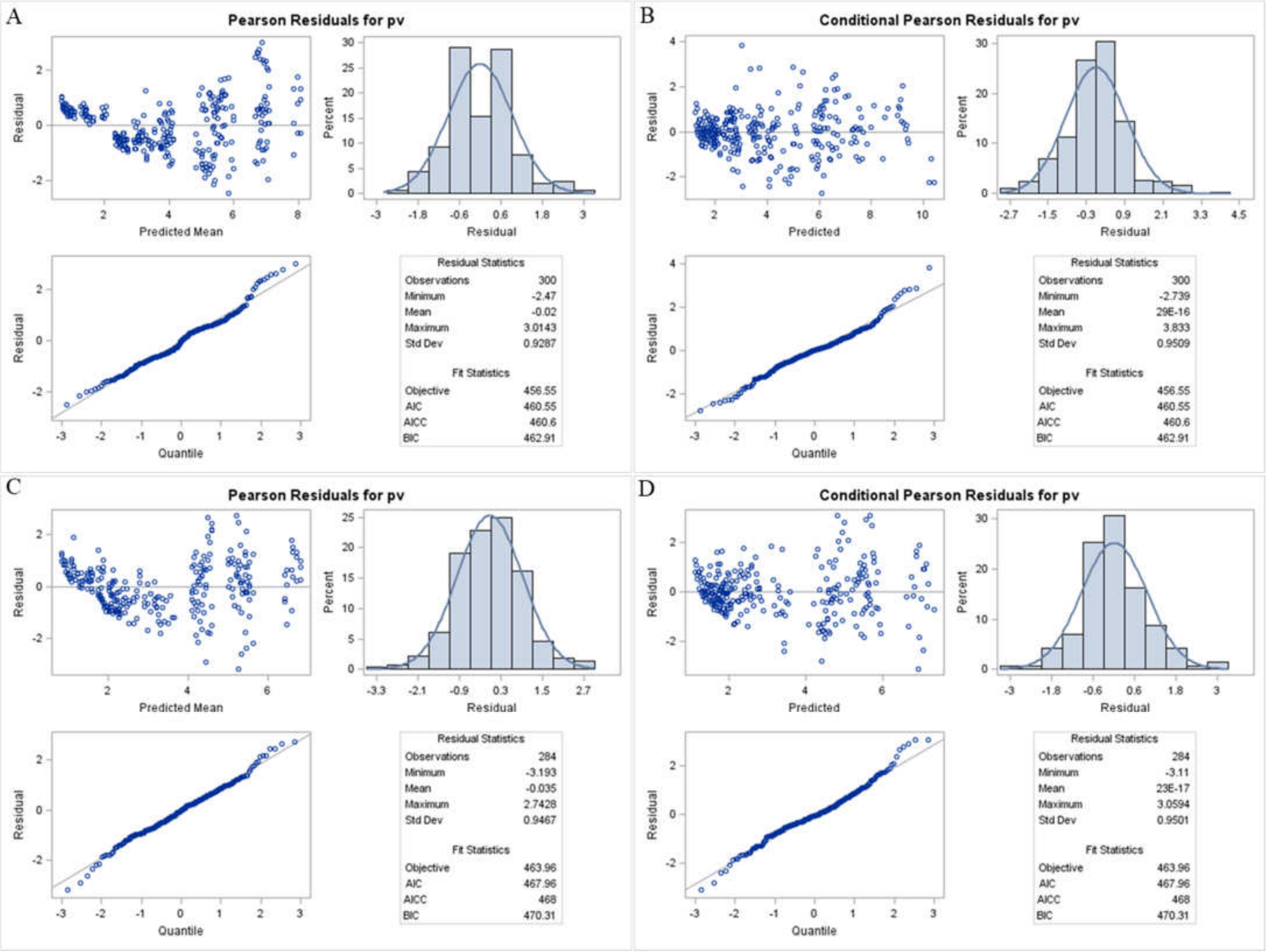

Supplement: Supplementary file 12 — Additional file 12. Examinations of the assumptions – normality and homoscedasticity of errors for the final mixed effects models derived from voluntary GZ (panels A and B) and CD (panels C and D) pseudo-panel datasets. Panels A and C present marginal Pearson residual plots, which are for the consideration of fixed effects only. Panels B and D visualize conditional Pearson residual plots, which are for the consideration of both fixed and random effects. The plot residuals vs. predicted outcome mean is used for examining the homoscedasticity assumption whereas the plots percent vs. residual and residual vs. quantiles are used for visualizing the normality assumption. The variable “pv” represents outcome, i.e., average units of platelet per donor. [file 12889_2021_10819_MOESM12_ESM.pdf]
