# Supplementary material for: The apheresis platelet donation was increased after a nationwide ban on family/replacement donation in China
Source: BMC Public Health. 2021 Apr 29;21:819. doi: 10.1186/s12889-021-10819-4 (PMC8082857; doi:10.1186/s12889-021-10819-4)
Supplement: Supplementary file 13 — Additional file 13. Five-fold cross-validation for the final models of voluntary pseudo-panel datasets. [file 12889_2021_10819_MOESM13_ESM.pdf]

Additional file 13. Five-fold cross-validation for the final models of voluntary pseudo-panel datasets.

	Voluntary GZ Subset	Voluntary CD Subset
RMSE1	1.1226	0.6458
RMSE2	0.8873	0.6756
RMSE3	1.0672	0.7114
RMSE4	1.0631	0.5327
RMSE5	0.8710	0.6045
Average $RMSE_{(cross\text{-}validation)}$ [a]	1.0313	0.6370
$RMSE_{(full\ sample)}$	0.9856	0.6329
ΔRMSE [b]	0.0457	0.0041
% change in RMSE [c]	4.6368	0.6478

[a] Average $RMSE_{(cross\text{-}validation)} = SQRT((RMSE_1^2 + RMSE_2^2 + \ldots + RMSE_5^2)/5)$.
[b] $\Delta RMSE$ = Average $RMSE_{(cross\text{-}validation)} - RMSE_{(full\ sample)}$.
[c] %change in RMSE = $\Delta RMSE \times 100 / RMSE_{(full\ sample)}$.
